# Game Intensity Analysis of Elite Adolescent Ice Hockey Players

**DOI:** 10.2478/hukin-2014-0126

**Published:** 2014-12-30

**Authors:** Arkadiusz Stanula, Robert Roczniok

**Affiliations:** 1 Department of Statistics, Methodology and Informatics, The Jerzy Kukuczka Academy of Physical Education, Katowice, Poland.

**Keywords:** thresholds, game analysis, oxygen uptake

## Abstract

The purpose of this study was to determine ice-hockey players’ playing intensity based on their heart rates (HRs) recorded during a game and on the outcomes of an incremental maximum oxygen uptake test. Twenty ice-hockey players, members of the Polish junior national team (U18), performed an incremental test to assess their maximal oxygen uptake (V̇O_2_max) in the two week’s period preceding 5 games they played at the World Championships. Players’ HRs at the first and second ventilatory thresholds obtained during the test were utilized to determine intensity zones (low, moderate, and high) that were subsequently used to classify HR values recorded during each of the games. For individual intensity zones, the following HRs expressed as mean values and as percentages of the maximal heart rate (HRmax) were obtained: forwards 148–158 b·min^−1^ (79.5–84.8% HRmax), 159–178 b·min^−1^ (85.4–95.6% HRmax), 179–186 b·min^−1^ (96.1–100.0% HRmax); defensemen 149–153 b·min^−1^ (80.0–82.1% HRmax), 154–175 b·min^−1^ (82.6–94.0% HRmax), 176–186 b·min^−1^ (94.5–100.0% HRmax). The amount of time the forwards and defensemen spent in the three intensity zones expressed as percentages of the total time of the game were: 54.91 vs. 55.62% (low), 26.40 vs. 22.38% (moderate) and 18.68 vs. 22.00% (high). The forwards spent more time in the low intensity zone than the defensemen, however, the difference was not statistically significant. The results of the study indicate that using aerobic and anaerobic metabolism variables to determine intensity zones can significantly improve the reliability of evaluation of the physiological demands of the game, and can be a useful tool for coaches in managing the training process.

## Introduction

Ice hockey is an intermittent high intensity body-contact team sport that requires a combination of aerobic and anaerobic fitness to perform a sequence of well-coordinated activities and a high level of technical skills (Montgomery, 1988; Cox et al., 1995; Roczniok et al., 2013). Performance in a variety of intermittent team sports has been related to the participant’s speed, power, strength, agility, and sustained ability to repeat short, high intensity bouts of activity throughout a match, rather than the capacity to sustain a steady submaximal work rate (Bangsbo et al., 1991; Stanula et al., 2014). Detailed knowledge of the activity profile and how it changes over a typical match would be helpful to coaches not only in order to enhance collaboration between players during technical-tactical combinations, but also to optimize techniques and training to sustain performance through the final minutes of play (Hamilton et al., 1991; Hoff et al., 2002). The extremely variable nature, intensity, and duration of activity encountered in ice hockey make it difficult to precisely determine the metabolic demands of this sport.

Many authors have attempted to quantify metabolic demands of ice hockey either using the time-motion analysis (TMA) (Bracko, 1992; Green et al., 1976; Lafontaine et al., 1998; Lothian and Farrally, 1994; Montgomery, 1988; Peddie, 1995) or measuring heart rates (HRs) and postperiod blood-lactate (BLa) values (Bracko et al., 1998; Noonan, 2010; Patterson et al., 1977; Spiering et al., 2003). The use of the TMA method is criticized, however, particularly in case of sport disciplines where effort intensity and duration are very irregular, such as in ice hockey, futsal or rugby (Duthie et al., 2003a). Some tactical movements ice-hockey players perform (body checking, starts and stops, rapid changes in skating direction, or shots on goal, etc.) are too short to be recorded, so their numbers are only added up or their total duration (Bracko, 1992; Peddie, 1995). This approach distorts information about their duration and intensity, leading to inaccurate estimates of the demands on the efficiency of the players’ physiological mechanisms. The quantification of metabolic demands by means of BLa has its weak points too. For instance, players’ BLa can only be measured during the breaks, because taking blood samples is practically impossible when the game is on. The length of time after which blood is sampled after high intensity exercise is of major significance; if it is too long blood lactate can be metabolized before measurements and consequently the demands of ice-hockey matches will be underestimated (Duthie et al., 2003b). Monitoring the ice-hockey players’ HR to estimate exercise intensity is also affected by some limitations. Individual differences in fitness levels and variations in exercise economy among ice-hockey players may make the researcher using this method draw incorrect conclusions about exercise intensity (Achten and Jeukendrup, 2003). Furthermore, generalizing intensity zones determined from the HRmax for all players may not be the most accurate approach to determine different intensity zones (Hills et al., 1998).

Being aware of the above limitations affecting the evaluation of the metabolic profile of exercise in team games performed using BLa and the HR as well as the TMA, researchers have put forward a method that analyses subjects’ HRs recorded during a standard incremental exercise test performed to volitional exhaustion together with V̇O_2_, V̇_E_, and V̇CO_2_. Changes in V̇O_2_ and V̇_E_ are used to determine two physiological points in gas exchange: the aerobic threshold/ventilatory threshold (V̇T_1_) and the anaerobic threshold/respiratory compensation point (RCP) (Foster and Cotter, 2006). The HR recorded at exercise intensity below V̇T_1_, between V̇T_1_ and RCP, and above RCP allows to divide exercise intensity zones into low, moderate and high. This approach has been found useful in evaluating the metabolism of athletes competing in individual sports, such as cross country running (Esteve-Lanao et al., 2005), skiing (Seiler and Kjerland, 2006) or road cycling (Lucia et al., 1999). As far as team sports are concerned, the V̇O_2_-to-V̇_E_ ratio determined during an incremental test for the purpose of evaluating exercise intensity during a game has been adapted for the needs of rugby union by Sparks and Coetzee (2013).

The purpose of this study was to determine ice-hockey intensity during games played by adolescent elite players based on their heart rates and the results of graded maximal test values. Its results may help coaches and players determine actual demands during a game. The information provides a starting point for programming of training activities in line with actual metabolic demands of a game situation.

## Material and Methods

### Subjects

The experiment involved 20 youth ice-hockey players (12 forwards and 8 defensemen), members of the Polish male national under 18 years team (U18). The analyzed data were recorded in subjects who participated in all periods of the 5 analyzed games at the World Championships, and performed an incremental exercise test for evaluation of V̇O_2_max. The test was carried out 2 weeks before the World Championships. Each subject was asked to refrain from using any ergogenic substances for 48 hours before the test, and from any physical activity which might affect their physical or physiological reactions. Ice-hockey players that failed to meet any of the requirements were not considered for the study. The accepted subjects were healthy, injury-free, fully rested, and adequately hydrated. Written informed consent was received from all participants and parents after a brief but detailed explanation of the aims, benefits, and risks involved with this investigation. The research project was approved by the Bioethics Commission at the Regional Medical Chamber in Cracow. The characteristics of ice-hockey players participating in the study are presented in Table 1. Standard Incremental V̇O_2_max test to the Point of Exhaustion.

A standard incremental maximal oxygen uptake test was conducted in the laboratory by means of open-circuit spirometry and computerized instrumentation. Each subject performed the test until voluntary exhaustion on a cycling ergometer platform Cyclus 2 (RBM elektronik-automation GmbH, Leipzig, Germany). Prior to the test, the players warmed up on the ergometer for 5 min of continuous pedaling at a power level of 1 W·kg^−1^ after which exercise intensity was increased to 4 W·kg^−1^ for a duration of 10 s. The first 3 min of the test were completed at an intensity of 1 W·kg^−1^ of body weight, and then intensity was increased every 3 min by 0.5 W·kg^−1^ of body weight. Exhaled air was continuously sampled by a gas analyzer K4 b^2^ (Cosmed, Italy) and the rate of oxygen uptake (V̇O_2_), carbon dioxide production (V̇CO_2_), minute ventilation (V̇_E_), and the respiratory exchange ratio (RER) were recorded every 5 s by an on-line computer system. The K4 b^2^ was calibrated in accordance with the manufacturer’s specifications at the beginning of each test day. The test was stopped if the subject failed to maintain a given RPM or if the V̇O_2_max criteria were met (e.g. RER greater than 1.10 at test termination; oxygen uptake reaching a plateau or starting to fall even though the work rate kept increasing or the maximal age-specific heart rate was reached) (Davis, 2006; McArdle et al., 2001). Throughout the test, HRs were recorded every 5 s by means of a Fix Polar Heart Rate Transmitter Belt (Polar electro OY, Kempele, Finland).

### V̇T_1_ and RCP

Two physiological gas exchange points were identified. The V̇T_1_ was determined using the criteria of an increase in V̇_E_/V̇O_2_ with no increase in V̇_E_/V̇CO_2_ and departure from the linearity of V̇_E_. The RCP was taken at a point corresponding to an increase in both V̇_E_/V̇O_2_ and V̇_E_/V̇CO_2_ (Chicharro et al., 2000). Two independent experienced researchers visually detected V̇T_1_ and RCP. The different gas exchange phases were used to determine HRs corresponding to 3 exercise intensities (Chicharro et al., 2000). HRs at exercise intensities below V̇T_1_ were classified as the low-intensity HRs; HRs at exercise intensities between V̇T_1_ and RCP were classified as moderate-intensity HRs; and HRs at exercise intensities above RCP were classified as high-intensity HRs (Lucia et al., 1999).

### Recording the subjects’ HR during ice-hockey games

Individual players’ HRs were recorded at 5 s intervals during 5 games played at the IIHF World Championship U18 using the Polar Team system (Polar Electro OY, Kempele, Finland). In the locker room, before a warm-up for the game commenced, a member of the research team helped each player to attach a Polar Team transmitter to their chests, making sure that the electrodes correctly contacted the skin at the lower sternum level. The transmitters were returned as soon as the game was over and the researcher transferred the recorded HRs via the Polar ProTrainer software (v.5) to the PC. The total time of the game was defined as including all stoppages, but excluding the regular breaks between periods.

### Statistical analysis

All data were stored and organized with the Microsoft Excel 2010 worksheet (Microsoft Corporation, Redmond, WA, USA). A special Visual Basic macro was used to divide HR values recorded during the games among three intensity zones (low, moderate and high) determined based on the incremental V̇O_2_max test. As a result, the time each player spent in each of the zones could be estimated and expressed as percentages of the total time of the game (counted without the regular breaks between periods). In the next step, the basic measures of descriptive statistics were calculated for each variable, i.e. means, minimum values, maximum values, and standard deviations). The statistical significance of the differences between the lengths of time the players spent in individual periods in each zone was determined with a one-factor analysis of variance, with the p level set at ≤0.05. The calculations were performed with the Statistica 10 statistical software package (StatSoft, USA).

## Results

Table 2 shows the values of parameters recorded during the incremental test and indicators derived from them according to the player’s position. The mean values of V̇O_2_max and the HRmax in the group of forwards were, respectively, 60.3 ± 5.0 ml·kg^−1^·min^−1^ and 186.3 ± 6.3 b·min^−1^, and in the group of defensemen 58.8 ± 8.7 ml·kg^−1^·min^−1^ and 186.4 ± 11.2 b·min^−1^. In the first group, the gas exchange threshold, V̇T_1_, was determined at an average HR of 159.0 ± 10.9 b·min^−1^, a value corresponding to 84.3% of HRmax; for the defensemen, the value was 154.0 ± 4.9 b·min^−1^, an equivalent of 81.5% of HRmax. The forwards’ RCP was established at an average HR of 178.0 ± 8.3 b·min^−1^, i.e. 95.1% of HRmax, whereas defensemen reached their RCP at an average HR of 175.2 ± 10.5 b·min^−1^, i.e. 93.2% of HRmax. Based on HR values corresponding to V̇T_1_ and RCP, three intensity zones called low, moderate and high were determined: 148–158 b·min^−1^, 159–178 b·min^−1^ and 179–186 b·min^−1^ for the forwards and 149–153 b·min^−1^, 154–175 b·min^−1^ and 176–186 b·min^−1^ for the defensemen.

### Game analysis

The mean values of HRs recorded in the forwards and defensemen during all analyzed matches were, respectively, 161 b·min^−1^ and 158 b·min^−1^, what corresponds to moderate intensity determined during the incremental test. However, the means of HRmax values recorded during the games in both the forwards and the defensemen (195.4 ± 9.9 b·min^−1^ and 195.6 ± 9.0 b·min^−1^, respectively) were greater than those derived from HRmax obtained in the incremental test (186.3 ± 6.3 b·min^−1^ and 186.4 ± 11.2 b·min^−1^). In ice-hockey, the regular time of play lasts 60 minutes of effective competition. From all analyzed games, it results that the forwards played an average of 1:18:28 ± 11:40 (h:mm:ss) and the defensemen 1:23:37 ± 09:38 (h:mm:ss). The detailed results summing up the analysis of the games are presented in Table 3.

**Figure 1:**
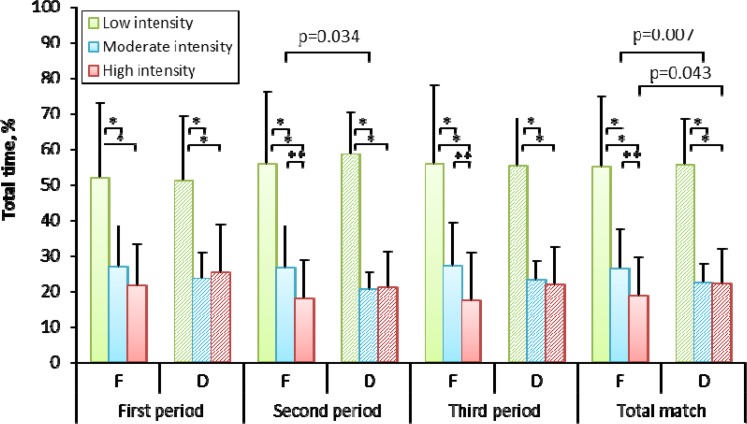
The time the players spent in different intensity zones as a percentage of the duration of the match and of individual periods (* − p≤0.001; ** − p≤0.05).

Figure 1 shows the average time the forwards and defensemen spent in particular intensity zones as percentages of periods and of the entire match. In each period, both formations stayed definitely longer in the low-intensity zone (over 50%). As far as the forwards are concerned, the time they spent in this zone increased with successive periods, by 51.86%, 55.63% and 55.77%. The longest time the defensemen spent in the low-intensity zone was in period 2 (58.37%). In terms of the entire match, the mean percentage time both formations spent in the low-intensity zone was similar: 54.91% and 55.62% for the forwards and the defensemen, respectively. The differences between the amounts of time the forwards and defensemen spent in this zone in each period and during the entire match were not statistically significant. The forwards spent less time in the high-intensity zone in each period compared with the defensemen: 21.49 vs. 25.27%, 17.91 vs. 21.04%, and 17.18 vs. 21.77% in periods 1, 2 i 3, respectively. As far as the entire match is concerned, the difference in the length of time the forwards and the defensemen played with high intensity was 3.32% and was statistically significant (p=0.043). A reverse tendency was observed when both formations were analyzed for the amount of time spent in the moderate intensity zone; in this case, the difference was the biggest in period 2 (5.86%; p=0.034). When the forwards and the defensemen were compared for the amount of time they spent in each of the intensity zones, then periods 2 and 3 turned out to be significantly different regarding the times the two formations spent in the low intensity and moderate intensity zones and in the low and high intensity zones (p<0.001). The forwards were also found to spend significantly (p<0.05) different amounts of time in the moderate and high intensity zones.

**Figure 2:**
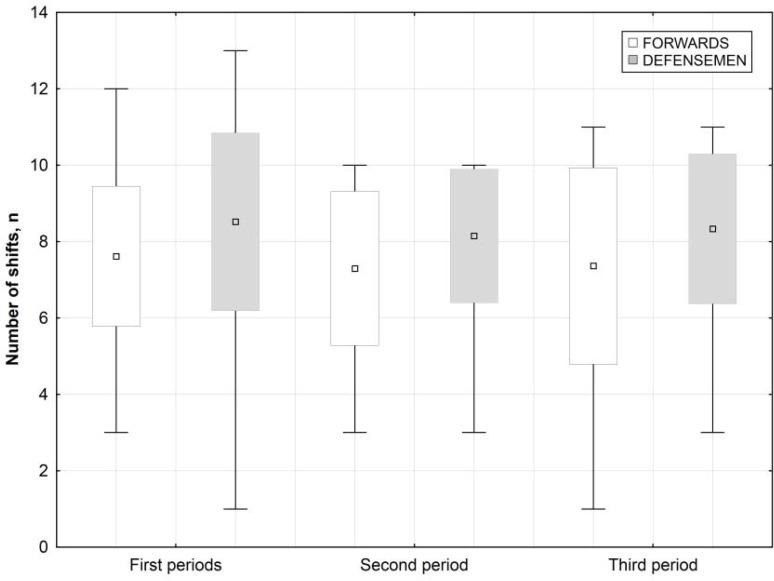
The number of shifts by formation during successive periods of a game. Values are given as Mean ± SD and Min–Max

Table 4 shows the mean lengths of the time that the forwards and defensemen spent in each intensity zone in periods and during the whole match. From the data it follows that over the whole length of the game the defensemen spent much more time in the low-intensity zone (46:08 vs. 42:34 mm:ss) and the high-intensity zone (18:38 vs. 14:58 mm:ss) than the forwards. At the same time, the forwards stayed longer in the moderate-intensity zone (20:57 mm:ss) than the defensemen (18:51 mm:ss). Figure 2 shows the number of shifts for both formations during a match. Because the defensemen were fewer (n=8), they were replaced in each period more often than the forwards (n=12). The mean numbers of forwards’ and defensemen’s shifts in particular periods were 7,61 vs. 8,52; 7,30 vs. 8,15 and 7,36 vs. 8,33, respectively. It is interesting to note that the number of shifts was highest for both forwards and defensemen in period 1 (max=12 and max=13).

## Discussion

Numerous studies point to a discrepancy between training intensity and the intensity of physical activity in a game situation (Cox, 1993; Cox et al., 1995; Daub et al., 1983; Green et al., 1976; Minkoff, 1982; Patterson et al., 1977; Quinney et al., 1982; Spiering et al., 2003). What follows from the observation is that a prerequisite to programming a training process is the availability of a quick, simple and most of all reliable analysis of the actual impacts of training and game demands on athletes (Szmatlan-Gabryś et al., 2006). The relevant method assigns the metabolic exercise characteristics determined from the ratio between the values of respiratory variables (V̇O_2_, V̇_E_, V̇CO_2_) recorded during an incremental test (Sparks and Coetzee, 2013) to HR values.

The main finding of this study is that ice-hockey players’ HRs recorded during a game and those obtained from an incremental test can be applied to estimate exercise intensity. It was also found that between the start and end of each period both forwards and defensemen spent most time in the low-intensity zone. In periods 1 and 3, the forwards spent slightly more time in that zone than the defensemen, but in the second period a reverse situation was observed. This finding is not surprising, because the analysis was based on HR values recorded between the start and end of each period, so both game stoppages and bench time were included. It is interesting to note that while in periods 1 and 3 the mean shift was clearly higher for the defensive players, in period 2 only a slight difference was noted (7.3 for the forwards and 8.15 for the defensemen). The results of this study are consistent with those obtained by Thoden and Jette (1975) who found that the junior offensive players spent less time on ice in each period than the defensemen by an average of 80 s. Even bigger differences were established for forwards in the professional NHL league, where the mean difference was 100 s. Léger (1980) noted a similar pattern. Having applied the TMA to a group of 80 juniors, Léger (1980) found that the forwards had spent on ice an average of 30 min, of which 18 min were active time and 12 min were consumed by stoppages. The defensemen spent on ice an average of 32 min, i.e. by 7.2% more than the forwards did; their active engagement in the game was 19.4 min while stoppages accounted for 13 min.

In the study, the percentage time the forwards and the defensemen spent in the moderate and high intensity zones was also established. In each period, the defensemen spent around 22% of their playing time in the high-intensity zone (HR exceeding 94.5% of HRmax) and approximately 22% in the moderate-intensity zone (HR between 82.6 and 94.0% of HRmax). In total, they spent more time in these two zones than the forwards for whom the respective values were around 19% (HR higher than 96.1% of HRmax) and 26% (HR from 85.4 to 95.6% of HRmax). The difference between the time the forwards and the defensemen spent in the high-intensity zone during the entire match was statistically significant (p=0.043).

As already mentioned in the introduction, most authors studying exercise intensity during a game used the TMA combined with observation and athletes’ HRs, which were subsequently related to the HRmax recorded during a game or an incremental exercise test. The novel analysis presented in this article, which was undertaken to quantify intensities in ice hockey, determines exercise intensity zones using physiological markers (V̇T_1_ and RCP) obtained during an incremental test. Its results are consistent with those published by other authors investigating the same topic. The results published by Green et al. (1976), Montgomery (1988) and Patterson et al. (1977), show that the mean HR of ice-hockey players actively engaged in the game ranges from 85 to 90% of HRmax, frequently exceeding 95%. Spiering et al. (2003) reported that the intensity of play during women’s national hockey competitions evaluated against the recorded HR values amounted to 90% of HRmax. The researchers concur that the intensity of work performed by defensemen and forwards on ice is equal in both formations. Based on the data collected by Green et al. (1976), differences were noted in HRs between defensemen and forwards. In another study by Green et al. (1978), there was no discrepancy in the average on-ice heart rates between two varsity forwards and defensemen. Paterson (1977) reported a similar heart rate difference between forwards and defensemen, except for a higher recovery heart rate for the defensive position. Peddie (1995) who studied varsity ice-hockey players during games also found the forwards’ and defensemen’s HR to be equal, amounting to an average of 82.5% of the HRmax.

From the physiological perspective, an incremental test with a cycling ergometer may not be the best way to determine HR intensity zones for the purpose of quantifying exercise intensities involved in an ice-hockey game (Akubat and Abt, 2011). Requiring the players to perform frequent accelerations, stops and changes in skating direction, ice-hockey is extremely diverse in terms of its nature, intensity and the duration of players’ movements. Moreover, the players frequently push to the limits the rules allowing body checking. Aggressive clashes strongly stimulate the nervous system, which contributes to increased release of adrenaline and thereby to a higher HR. Compared with that, cycling on the ergometer is a continuous activity performed with steady velocity (Stanula et al., 2013). This implies that metabolism of the muscles activated in a game situation is different than that during a cycling ergometer test, which explains why the means of the HRmax recorded during these two physical activities are different too.

## Study limitation

The telemetric measurement of the HR used to evaluate the aerobic demands on ice-hockey players has certain shortcomings that must be accounted for when interpreting the results. In a game situation, the players’ HR may be affected by many factors, also other than those related to increased oxygen cost, such as emotions, static work of the torso muscles, varying demands of the play, torso temperature elevated by the player’s outfit that reduces the amount of heat transferred from the body (Montgomery, 1988).

## Conclusions

A method that uses the aerobic and anaerobic metabolism variables to determine exercise intensity zones can significantly improve the reliability of assessment of the physiological demands of the game, which makes it a very useful tool for sports scientists and other sport-related professionals in managing the training process. It can replace the approach where training intensity zones are derived from the HRmax or from intensities that have been assigned a priori to training loads. The method presented in this article provides coaches with a tool for creating an inventory of training loads and player’s activities during a game, consistent with their motor preparation. This universal approach has already been tested for ice-hockey, a sport discipline characterized by intermittent physical activity of varying intensity, where the intervals are not long enough to ensure full recovery.

## Figures and Tables

**Table 1:** Physical characteristics of the ice hockey players taking part in the investigation

Variables	Subjects (n=20)	Range
Age (yrs)	16.7 ± 0.75	15.0–18.0
Body height (cm)	182.1 ± 5.45	174.0–191.0
Body mas (kg)	79.0 ± 7.16	66.0–94.5
BMI (kg·m^−2^)	23.8 ± 1.86	21.5–27.5

**Table 2:** The results of the incremental test by the player’s position in the game. Values are given as Mean ± SD

**Parameters**	**Forwards (n=12)**	**Range**	**Defensemen (n=8)**	**Range**
V̇O_2_max (ml·kg^−1^·min^−1^)	60.3 ± 5.0	53.0–69.0	58.8 ± 8.7	46.0–73.0
HRmax (b·min^−1^)	186.3 ± 6.3	177.0–201.0	186.4 ± 11.2	166.0–204.0
RERmax	1.62 ± 0.10	1.43–1.77	1.57 ± 0.24	1.06–1.89
V̇T_1_ (%V̇O_2_max)	69.1 ± 8.1	56.4–79.4	63.4 ± 4.7	59.9–73.1
V̇T_1_ (ml·kg^−1^·min^−1^)	41.6 ± 5.3	31.0–48.0	37.2 ± 5.8	27.7–47.3
V̇T_1_ (RER)	0.95 ± 0.02	0.92–0.98	0.94 ± 0.04	0.88–1.00
V̇T_1_ (b·min^−1^)	159.0 ± 10.9	146.0–178.0	154.0 ± 4.9	146.0–160.3
V̇T_1_ (% HRmax)	84.3 ± 4.5	77.8–92.4	81.5 ± 4.9	75.9–88.6
RCP (%V̇O_2_max)	85.5 ± 6.8	74.8–95.7	81.2 ± 9.2	62.3–90.6
RCP (ml·kg^−1^·min^−1^)	51.6 ± 5.6	39.7–57.7	47.2 ± 5.1	41.7–56.3
RCP (RER)	1.05 ± 0.03	1.02–1.11	1.07 ± 0.03	1.03–1.10
RCP (b·min^−1^)	178.0 ± 8.3	166.0–193.0	175.2 ± 10.5	162.7–190.7
RCP (% HRmax)	95.1 ± 2.4	91.5–98.9	93.2 ± 4.3	87.7–98.9

Low-intensity heart rate zone (b·min^−1^) [% HRmax]	148–158 [79.5–84.8]	135–177	149–153 [80.0–82.1]	141–159
Moderate-intensity heart rate zone (b·min^−1^) [% HRmax]	159–178 [85.4–95.6]	146–193	154–175 [82.6–94.0]	146–191
High-intensity heart rate zone (b·min^−1^) [% HRmax]	179–186 [96.1–100.0]	166–201	176–186 [94.5–100.0]	163–204

V̇O_2_max — maximum oxygen uptake; HRmax — maximum heart rate; RERmax — maximum respiratory exchange ratio; V̇T_1_ (%V̇O_2_max) — ventilatory threshold expressed as a percentage of the maximum oxygen uptake; V̇T_1_ (ml·kg^−1^·min^−1^) — oxygen uptake at the ventilatory threshold; V̇T_1_ (RER) — respiratory exchange ratio at ventilatory threshold; V̇T_1_ (b·min^−1^) — heart rate at the ventilatory threshold; V̇T_1_ (% HRmax) — ventilatory threshold expressed as a percentage of the maximum heart rate; RCP (%V̇O_2_max) — respiratory compensation point expressed as a percentage of the maximum oxygen uptake; RCP (ml·kg^−1^·min^−1^) — oxygen uptake at the respiratory compensation point; RCP (RER) — respiratory exchange ratio at the respiratory compensation point; RCP (b·min^−1^) — heart rate at the respiratory compensation point; RCP (% HRmax) — respiratory compensation point expressed as a percentage of the maximum heart rate; %HRmax — percentage of the maximum heart rate.

**Table 3:** The descriptive statistics of variables recorded during matches

Variable	Formation	Mean ± SD	Range
HRmax (b·min^−1^)	F	195.4 ± 9.9	177–224
	D	195.6 ± 9	173–213
HRavg (b·min^−1^)	F	160.9 ± 6.5	148–176
	D	157.9 ± 5.7	147–169
Game time (h:mm:ss)	F	1:18:28 ± 11:40	49:00–1:34:30
	D	1:23:37 ± 09:38	53:20–1:36:45

HRmax — maximum heart rate; HRavg — average heart rate

**Table 4:** Descriptive statistics of individual intensity zones during games

Variable	Formation	Low	Moderate	High
Total time spent in the intensity zones during the first period (mm:ss)	All	13:43 ± 06:33	06:39 ± 03:05	05:58 ± 03:29
	F	13:26 ± 06:48	06:48 ± 03:26	05:37 ± 03:21
	D	14:09 ± 06:12	06:23 ± 02:28	06:31 ± 03:41
Total time spent in the intensity zones during the second period (mm:ss)	All	15:51 ± 05:11	06:52 ± 03:05	05:31 ± 03:26
	F	15:18 ± 05:54	07:22 ± 03:37	05:06 ± 03:23
	D	16:45 ± 03:40	06:02 ± 01:44	06:11 ± 03:26
Total time spent in the intensity zones during the third period (mm:ss)	All	14:22 ± 06:04	06:39 ± 03:09	04:53 ± 03:29
	F	13:50 ± 06:43	06:47 ± 03:45	04:15 ± 03:35
	D	15:14 ± 04:48	06:26 ± 01:53	05:56 ± 03:08
Total time spent in the intensity zones during the entire match (mm:ss)	All	43:55 ± 13:54	20:09 ± 08:21	16:21 ± 09:30
	F	42:34 ± 15:31	20:57 ± 09:47	14:58 ± 09:26
	D	46:08 ± 10:40	18:51 ± 05:12	18:38 ± 09:19
